# Some Points in Oral Surgery of Interest to the General Practitioner

**Published:** 1883-12

**Authors:** George L. Parmele


ARTICLE IV.

### SOME POINTS IN ORAL SURGERY OF INTEREST TO THE GENERAL PRACTITIONER.—(Continued.)

BY GEORGE L. PARMELE, M. D., D. D. S.,

(Presented to the Ninety-Second Annual Meeting of the Connecticut Medical Society, May 23, 1883.)

They usually do not recognize the fact that, as a general thing, decayed teeth, teeth with inflamed alveolus, with matter exuding from around the teeth, are the means of producing more nervous disorders, more terrible consequences to the general health, than almost any other thing that can happpen." . . . . . . . Some simple knowledge of this subject would enable the medical man to treat them more successfully than he docs. It is a matter of regret that medical men generally have so little knowledge of this subject." He related numerous cases to illustrate his views.

At the same meeting Dr. Frank Hamilton expressed similar views.

Samuel Sexton, M. D., Surgeon to the New York Ear Dispensary, in the *American Journal of Medical Science*, says: "The apathy which has always existed on the part of the profession regarding this subject has left the treatment of diseases of the teeth in the hands of men who have occupied themselves almost exclusively with its mechanical department, and who, as a rule have but little to do with the teeth in a medical aspect. It is greatly to be regretted that a field of such interest has been abandoned by the profession. Many affections of the teeth lead to most grave and intractable diseases of the regions presided over by the sympathetic system, which are often suffered to be long unattended before they are brought under appropriate management. Thus an ear, eye, or throat difficulty may become firmly seated, or a neuralgia, which renders life intolerable, established. When I look back at the operation for the removal of Meckel's gangloin, which I twice witnessed, for the relief of facial neuralgia, it occurs to me that the most simple of remedies could have controlled that disease when it was first induced, as was probable in these instances, by a carious tooth. I think you will find Dr. Sexton's article well worthy of perusal.

I cite the following case from my own practice—a simple one—as showing long continued unsuccessful treatment of neuralgia of about five years standing, of the trifacial nerve, caused by a periodontal abscess opening into the antrum, and causing severe neuralgia, with the absence of pain in the teeth, which caused the trouble,

Mrs. H., aged 50, was referred to me September, 1881, by Dr. C., for treatment. I found the left superior canine with its crown missing, and the root loose from chronic periodontis. The bicuspid and first molar were missing, and half an inch above the margin of the gum, at a point formerly occupied by the second bicuspid, a fistulous opening was discovered, which, upon probing, was found to lead to the antrum. Removal of the canine root was followed by a single discharge of pus, and a probe passed into its socket also entered the antrum. Surrounding each of these openings was carious bone, greater in amount, however, at the canine opening where the maxilla was dissolved, for a space half an inch in diameter.

Having thoroughly removed all diseased bone with bone-cutting burs, in the dental engine, I then, by means of a small tube attached to a fountain syringe, allowed about a pint of warm salt water to flow into the antrum at the anterior opening, which made its exit at the posterior opening.

By changing the tube to the posterior opening, I reversed the current, thus insuring throughout cleaning of the sinus. This was followed by an injection of

Eucalypti (Sanders' Sons) ℨ i.

Iodoformi, gr. x.

Aquæ, ℥ j.

M.

Then a tent of candle-wicking saturated with glycerine and eucalypus, was passed into the antrum at the anterior, and brought out at the posterior opening where the two ends were tied together. A few days later floss silk was substituted for the wicking. This treatment continued a week, when the tent was omitted and the patient being instructed, from that time, kept the parts clean herself. In a month, after a few stimulating injections of

Zinci Sulphatis, gr. iij.
Plumbi Acetatis, gr. v.
Tincturæ Catechu, gtts. x.
Aquæ, ℥ i.
M., the parts had regained their normal condition, the neuralgia disappeared, and the openings gradually healed. The fountain syringe for thorough cleansing of the antrum I find very useful. I have never seen its use for this purpose mentioned, nevertheless it may be an old idea, though new to me. It is more easily managed, either by operator or patient, than any other form of syringe. I have always believed that dentistry ought never to have been established as a separate profession, but that any one desiring to practice in any department of medicine should be required to follow a regular medical education, and then to perfect himself in the desired specialty.

I think that a few lectures on dental pathology, introduced into our medical courses, would be of great value to the general practitioner. I desire to ask the co-operation of the medical profession in the preservation of the temporary and deciduous teeth. You come more in contact with the little ones, and have better chances of observing their mouths, and of giving advice to their parents than the dentist.

The deciduous teeth for the most part receivc attention only when the sufferings of the child render palliative treatment necessary. Perhaps you are not fully alive to the consequences, direct or indirect, of the premature loss of these organs upon the future health and comfort of the child.

On these teeth to a great deal depends, not only the regularly and usefulness of the permanent set, but the perfection of the whole physical organization. At no other time in life are sound, serviceable teeth more necessary as aids to digestion than during these years of growth and development. It is therefore a sad mistake when temporary teeth are permitted to be prematurely extracted. Serious may be the consequences when days and nights of odontalgic pain are suffered to elapse without mitigation. In fact the health and welfare of the individual may be seriously impaired by the neglect of the treatment which these teeth demand in the majority of cases. Parents have not been educated as to their value, and a great many dentists practically ignore the matter because of the difficulty and tediousness of operations on the teeth of young children, and because these cases do not directly pay pecuniarily.

I would not have you construe what I have said concerning premature extraction of these teeth in meaning that they should never be extracted, for oftentimes the removal of some of them is an absolute necessity, as for example, an alveolar abscess connected with the deciduous tooth not unfrequently results in necrosis of the surrounding bone, involving oftentimes the loss of the germs of several permanent teeth. The following case illustrates this point:

Johnnie F., of Irish parentage, and scrofulous diathesis, about five years of age was brought to me by his mother, who desired the extraction of the right inferior second molar to relieve pain, which he referred to that tooth. Examination showed the surrounding parts congested and highly offensive, but the tooth complained of was so slightly attacked by caries as to show at a glance that it was not the cause of the trouble. Traction upon it however, showed it, and a portion of the adjoining bone, quite loose and free from the maxilla. After making a slight incision I was able to gently lift out the exfoliated portion (which I pass around for your inspection). It will be observed that the germs of the permanent bicuspids are in the sequestrum, but on examination of the boy's mouth at the present time (it being now about seven years since I first saw him). shows the permanent canine also missing, the germ of which occupied the space where the most acute inflammation seems to have been. Owing to ignorance of the mother the history of the case is rather meager, she not being aware of any trouble until a day or two before consulting me. I am inclined to think, however, that the trouble resulted from a blow which the boy said he received about a year before. The only after-treatment required was thorough cleansing with carbolized water—the parts healing generally.

The whole of the time allotted to me for this essay could to advantage be used on this question alone, but I will tax your patience only long enough to again ask that you use your influence in trying to impress upon parents the necessity of having these teeth carefully and frequently examined by a competent practitioner, for more can be done to *avoid* impairment of the teeth before the age of twelve than at any other time.

While speaking of children, I will simply call your attention to a deformity of the oral and nasal cavities and the adjacent bones, which often seriously impairs mastication and speech, and occasions difficulty in nasal breathing, with its attendant ills. In this deformity, which is produced by the habit in the infant of thumb-sucking, there is a forward and upward projection of the superior maxilla, accompanied by a fan-like projection of the teeth and upper lips. The bones of the floor of the nares are elongated, narrowing the nasal cavity, and generally there is a lateral deviation of the bridge and septum of the nose which causes stenosis, from the thickening of the tissues. In the lower jaw a reversed deformity often exists which deranges the proper articulation of the teeth, interfering with mastication, and consequently impairs digestion. The practice of lip, finger, or tongue-sucking may be reckoned under this head, and can cause considerable deformity at an age when the parts are so easily moved and moulded, and whenever any of these pernicious habits have been formed in children, no pains should be spared to break them up. This habit apparently so innocent, is often encouraged by those ignorant of these consequences, as the peace and quiet given to parents while irritable infants are so engaged is considered especially desirable.

Although a habit hard to correct, even in quite young children, and one which waxes stronger with increased years, to break it up is easier than to remedy the resulting deformity. A night dress without sleeves, fitting tightly about the neck, inside of which the arms can move at will, is one good mode of treatment. A very interesting article on this subject will be found in the *Boston Medical and Surgical Jonrnal*, 1878, by T. H. Chandler, M. D.

Physicians and dentists have long observed that during pregnancy the teeth of females are particularly prone to decay. While I have never met with any theory as to the cause of such increase, I am inclined to the opinion that to the abnormal condition of the female pelvic organs, acting through the digestion, together with a vititated condition of the oral fluids at this time is due this result.

The same is true, to a great extent, of females suffering from uterine diseases. When such a condition exists, whatever the cases, the treatment should be of temporary nature, so as to lessen the strain on the nervous system of the patient, and because so-called permanent operations will, while the same predisposing causes exist to produce caries, be found soon to fail. Gutta-percha or oxyphosphate of zinc fillings will be found in such cases to outlast any metallic stoppings.

Having often been consulted by medical men as to the advisability of extracting teeth from the mouths of pregnant women, a few words here, on this topic, may not be out of place. While the extraction of a tooth is an operation of common occurrence, it should not be considered as trifling, for it produces invariably a sudden nervous impression—*shock*.

There is a rupture of from one to three square inches of living tissue, containing blood vessels, and from one to four nerves, while often there is more or less extensive fracture of the bony processes and profuse hemorrhage; and in the case of a pregnant woman, all these things should be borne in mind, together with her previous history and condition.

That which may be endured with impunity by the nervous system at one time, may at another be attended by serious injury and prostation. The condition of the tooth also should be taken into consideration, as to its amenability to temporary treatment for allaying pain incident to it, also to the amount of force required in its extraction, and the consequent shock.

Where a choice has to be made between allowing a tooth to remain, involving odontalgia, severe neuralgia, antral or alveolar abscess (conditions compromising the general health and comfort of the patient), and the removal of the offender, the latter course I should think the proper one to follow, but this contingency can usually be avoided, and does not often arise. It should be a choice between two evils, and especial care should be used to avoid, as far as possible, all nervous impressions. When extraction must be resorted to, I should advise the use of an anæsthetic, preferably nitrous oxide, there being less danger attending its administration, and because its effects so quickly pass away; and furthermore the operation had better be performed at the home of the patient, especially if she be in delicate health, so as to avoid the nervous irritability which is often awakened by seeing the paraphernalia of the extracting room. I should consider it reprehensible to extract teeth at this time to prepare the mouth for an artificial denture, as the operation should only be performed during pregnancy as the last resort.

As the greater danger of miscarriage exists in the earlier mouths of pregnancy, temporary treatment, if possible, should then be resorted to, if only for a few weeks. Still, where the tooth is not *easily* amenable to treatment, I believe that the shock from extraction would be less likely to do harm than the exhaustion produced by long continued pain.

In ending, I can truly say that I feel honored with my appointment as an essayist for this occasion, an honor which I desire to acknowledge.

